# Costunolide Reduces DN Inflammatory Response and Renal Thrombosis by Inhibiting NET Formation

**DOI:** 10.1155/jdr/1159325

**Published:** 2025-07-17

**Authors:** Xiangjing Wang, Lina Zhang, Ke Huang, Chengli Lou, Yuanying Xia, Yijing Zhou

**Affiliations:** ^1^Department of Nephrology, Jiaxing Hospital of Traditional Chinese Medicine, Jiaxing, China; ^2^Department of General Practice, Jiabei Street Community Health Service Center, Jiaxing, China

## Abstract

**Background:** Diabetic nephropathy (DN), a prevalent microvascular complication of diabetes, is characterized by chronic inflammation, oxidative stress, and renal thrombosis. This study is aimed at assessing the therapeutic effects of costunolide (COS) on DN and investigating its mechanism of action in reducing inflammation and platelet activation–mediated thrombosis by inhibiting the formation of neutrophil extracellular traps (NETs).

**Methods:** A DN mouse model was established using a high-sugar, high-fat diet combined with streptozotocin (STZ) administration, followed by treatment with varying doses of COS. The efficacy of COS was assessed through renal function indicators, including 24-h urinary protein levels, serum creatinine, and blood urea nitrogen, alongside renal histopathological analyses using hematoxylin-eosin, Masson's trichrome, and periodic acid–Schiff staining. Transcriptomic analysis was performed to identify gene expression changes in renal tissues after COS treatment. Based on transcriptomic findings, the impact of COS on inflammatory and platelet activation–related markers (IL-1*β*, IL-6, TNF-*α*, CCL2, and CD41) was further evaluated. Additionally, the expression of NET formation-related factors (MPO, CitH3, IGTAM, PAD4, C3, and fibrinogen) was analyzed using immunofluorescence, western blot, and ELISA. To validate the in vivo findings, isolated neutrophils were treated with COS in vitro to assess its inhibitory effects on NET formation, including markers such as SYTOX Green, CitH3, ROS, and PAD4.

**Results:** COS treatment significantly improved renal function and mitigated histopathological damage in DN mice. Transcriptomic analysis indicated that COS modulated pathways associated with inflammation and platelet activation, including the complement and coagulation cascades, biosynthesis of cofactors, cytokine–cytokine receptor interactions, NET formation, and NOD-like receptor signaling. COS markedly reduced the expression of inflammatory markers (IL-1*β*, IL-6, TNF-*α*, and CCL2) and the platelet activation marker CD41 in renal tissues. Moreover, COS decreased the expression of NET-related proteins, including MPO, CitH3, PAD4, IGTAM, C3, and fibrinogen, while lowering the CitH3/H3 ratio. In vitro, COS significantly inhibited PMA-induced NET formation in neutrophils, as evidenced by reduced SYTOX Green + CitH3^+^ expression and decreased levels of PAD4 and ROS.

**Conclusion:** COS alleviates inflammation and platelet activation–mediated thrombosis in DN mice, potentially by inhibiting excessive NET formation. These findings highlight the therapeutic potential of COS in managing DN.

## 1. Introduction

Diabetes mellitus is a chronic condition defined by impaired glucose metabolism, which can damage cardiovascular systems, retinal microvessels, and renal small blood vessels [1]. Among its complications, diabetic nephropathy (DN) stands as the most significant chronic microvascular disorder and a leading cause of end-stage renal disease [2]. The hallmark pathological features of DN include glomerular basement membrane thickening, tubulointerstitial fibrosis, and extracellular matrix accumulation. These changes are strongly associated with chronic inflammation and oxidative stress triggered by hyperglycemia [3]. In hyperglycemic conditions, reactive oxygen species (ROS) production increases, intensifying oxidative stress and noninfectious inflammatory responses in DN. Concurrently, hyperglycemia activates endogenous and exogenous coagulation pathways while reducing fibrinolytic activity [4]. These processes damage vascular endothelial cells, inducing hypercoagulability and venous thrombosis. Such events further exacerbate renal fibrosis and vascular injury in DN. As a result, effective management of chronic inflammation and intrarenal thrombosis has emerged as a pivotal goal and a major challenge in the therapeutic landscape of DN.

Neutrophil extracellular traps (NETs) are recognized as key inflammatory mediators in the pathological progression of DN [5]. NETs consist of a network of chromatin, histones, and various neutrophil granule proteins, such as myeloperoxidase (MPO) and NE. These structures are released by neutrophils in response to pathogen stimulation to capture and eliminate microbes. However, excessive NET formation can result in autologous tissue damage [6].

Studies have shown that NETs not only contribute to the inflammatory response but also play a critical role in thrombosis. NETs exacerbate the coagulation response by activating endothelial cells and monocytes, which promotes the formation of microthrombi [7, 8]. In DN, NETs are pivotal in glomerular injury and tubular fibrosis. They also exacerbate localized renal thrombosis by triggering inflammatory responses and damaging vascular endothelial cells, thus contributing to renal injury [9]. Furthermore, NET levels are significantly elevated in diabetic patients, suggesting their involvement in the pathological processes of diabetes, including glomerular thrombosis and inflammatory cell infiltration [10]. One study demonstrated that treatment of diabetic mice with GSK484, a pharmacological inhibitor of PAD4, significantly reduced NET markers (PAD4 and CitH3) in glomeruli and mitigated renal injury [11]. Therefore, inhibiting NET formation presents a promising therapeutic strategy for slowing the progression of DN, offering significant potential for clinical application.

Costunolide (COS) is a sesquiterpene lactone found abundantly in Asteraceae plants. It exhibits significant anti-inflammatory, antioxidant, and immunomodulatory activities [12, 13]. Previous studies have demonstrated that COS effectively reduces the expression of various inflammatory markers, such as TNF-*α*, IL-1*β*, and IL-6; alleviates oxidative stress; and provides protective effects in several inflammatory disease models [14]. Additionally, COS has shown potential in modulating coagulation, benefiting the vascular system by reducing platelet aggregation and inhibiting thrombosis [15, 16]. Despite these promising findings, COS has not been investigated in the context of diabetes mellitus and its associated renal complications. In this study, we first evaluated the therapeutic effect of COS on DN by establishing a DN mouse model. We then assessed the impact of COS on inflammation, intrarenal thrombosis, and NET formation in DN mice, integrating transcriptomic analysis. To further validate these findings, we cultured neutrophils in vitro to examine the inhibitory effect of COS on NET formation.

## 2. Materials and Methods

### 2.1. Reagents

The experimental reagents, pharmaceuticals, and other materials necessary for this study are described in detail in the supporting information.

### 2.2. Animals

We used 6–8-week-old healthy male C57BL/6 mice, weighing 20–22 g, for the experiments. These mice were purchased from Beijing Specific Biotechnology Co. Ltd (License No. SCXK [Beijing] 2024-0001). The mice were housed in groups of five per cage under controlled environmental conditions (24 ± 2°C, 55 ± 5% relative humidity, and a 12-h light–dark cycle) and had ad libitum access to food and water. All experimental procedures followed the Guidelines for Animal Ethics and received approval from Zhejiang Chinese Medical University (IACUC-20250217-07).

### 2.3. Animal Model Establishment and Grouping

After 1 week of acclimatization, 60 mice were randomly divided into two groups. The control (CON) group, consisting of 10 mice, was fed a normal diet, while the remaining 50 mice were given a high-sugar, high-fat diet. After 8 weeks on this diet, the modeling process began. The procedure, as previously described [17], involved a 12-h fasting period with no water. We then injected 30 mg/kg of STZ intraperitoneally into the experimental group, while the CON group received an equal volume of 1% sodium citrate buffer. Three days postinjection, we collected blood from the tail vein to measure random blood glucose levels. A glucose concentration ≥ 16.7 mmol/L was used as the criterion for the induction of Type 2 diabetes mellitus (T2DM). Following this, all mice continued their respective diets, and 24-h urine protein quantity (24 h-UTP) was measured weekly. DN modeling was confirmed by random blood glucose ≥ 16.7 mmol/L and 24 h-UTP ≥ 20 mg.

The 50 mice that successfully developed the DN model were randomly assigned into five groups, with 10 mice per group: the DN model group (DN), the irbesartan (IRB) positive CON group (DN + IRB), the low-dose COS group (DN + COSL), the medium-dose COS group (DN + COSM), and the high-dose COS group (DN + COSH). The CON and DN groups each received daily saline gavage (0.2 mL/kg), while the DN + IRB group was gavaged with 40 mg/kg of IRB daily [18]. The DN + COSL, DN + COSM, and DN + COSH groups were administered 10 mg/kg, 20 mg/kg, and 40 mg/kg of COS daily, respectively [19]. Each group received the corresponding treatment for 8 weeks. The CON group was fed a normal diet, while the other groups continued with a high-sugar, high-fat diet throughout the experiment. Blood glucose levels and body weight were monitored and recorded weekly. After treatment for 8 weeks, we collected 24-h urine samples from each group using metabolic cages. Blood was obtained via intraocular canthus sampling. Following euthanasia, we performed a necropsy, opening the abdominal cavity to collect the left kidney, which was fixed in 4% paraformaldehyde. The right kidney was snap-frozen in liquid nitrogen and stored at −80°C for further analysis.

### 2.4. Renal Function Index Test

We centrifuged the collected 24-h urine samples at 4000 rpm for 10 min and separated the supernatant. The 24 h-UTP in each group was measured following the kit instructions. Blood samples were centrifuged at 3500 rpm for 15 min, and the serum was collected. The serum levels of creatinine (Cr) and blood urea nitrogen (BUN) were then quantified according to the kit instructions.

### 2.5. Renal Histopathology and Morphology Detection

Renal tissues were extracted from the 4% paraformaldehyde solution, dehydrated, paraffin-embedded, and sectioned at 4 *μ*M thickness. The sections were stained with H&E, Masson, and PAS. After sealing the slides, we examined them under a microscope and captured images for analysis. H&E staining was used to assess the pathological morphology of renal tissues, and the scores were quantified. Masson staining was employed to evaluate renal fibrosis, while PAS staining was used to examine glycogen deposition. We quantified them using ImageJ software.

### 2.6. Transcriptomic Assay

Renal tissues were removed from −80°C, and total RNA was isolated. RNA integrity was assessed using the Bioanalyzer 2100 system (Agilent Technologies, California, United States). Messenger RNA was purified from total RNA using poly-T oligo-attached magnetic beads. After fragmentation, the first strand cDNA was synthesized using random hexamer primers, followed by the second strand cDNA synthesis using dUTP instead of dTTP. The directional library was prepared through end repair, A-tailing, adapter ligation, size selection, USER enzyme digestion, amplification, and purification. Library quality was assessed using Qubit and real-time PCR for quantification and the Bioanalyzer for size distribution detection. Libraries were pooled based on effective concentration and targeted data amount, then sequenced using the Illumina platform. The sequencing principle is “Sequencing by Synthesis,” where fluorescently labeled dNTPs, DNA polymerases, and adapter primers are added to the sequencing flow cell. As each sequencing cluster extends its complementary strand, the addition of each fluorescently labeled dNTP releases a corresponding fluorescence signal, captured and converted into sequencing peaks by the sequencer to obtain the target fragment sequence information.

Differentially expressed genes (DEGs) between the DN and CON groups, as well as between the DN + COSH and DN groups, were analyzed using DESeq2 and edgeR software. For DESeq2 with biological replicates, differential expression analysis was performed using the DESeq2 R package (1.20.0), which adjusts the resulting *p*_adj_ using the Benjamini and Hochberg method to control the false discovery rate. The threshold for significant differential expression was set at *p*_adj_ ≤ 0.05 and |log_2_(foldchange)| ≥ 1. For edgeR without biological replicates, read counts were adjusted using the edgeR R package (3.22.5) by scaling normalization factors to eliminate sequencing depth differences between samples, followed by differential expression analysis. The resulting *p*_adj_ was adjusted using the Benjamini and Hochberg method, with the same significance threshold. Finally, KEGG pathway enrichment analyses were conducted on the identified DEGs.

### 2.7. Enzyme-Linked Immunosorbent Assay (ELISA)

Frozen renal tissues were homogenized, and the expression levels of IL-1*β*, IL-6, TNF-*α*, CCL2, and fibrinogen in the tissue homogenates were measured using ELISA. The assay was performed according to the instructions provided with the corresponding kits.

### 2.8. Biochemical Assay

Frozen renal tissues were homogenized, and C3 levels were measured using a commercial kit. Additionally, cell samples from each group were collected and homogenized to detect ROS levels. For ROS measurements, ROS levels were normalized to the PMA group (cells permeabilized with 100 nM PMA for 3 h). ROS production was expressed as a fold change from baseline. All operations were carried out according to the operational instructions of the kits.

### 2.9. Isolation and Extraction of Mice Peripheral Blood Neutrophils

Neutrophils were isolated from the peripheral blood of SPF-grade healthy male C57BL/6 mice. Blood was collected in anticoagulated vacuum blood collection tubes, and neutrophils were separated using the Animal Peripheral Blood Neutrophil Separation Kit (LZS1091), following the manufacturer's instructions. Specifically, to isolate and purify neutrophils from a blood sample, first, add 5 mL of separation medium to a 15-mL sterile centrifuge tube, and then slowly add 2 mL of an 80% concentration separation medium solution (mixed with sample diluent in a 4:1 ratio) to form a stable gradient interface. Next, mix anticoagulated blood with red blood cell sedimentation liquid in a 2:1 ratio, and carefully layer it on top of the gradient interface, ensuring that the volume of separation medium is not less than that of the blood sample. After that, centrifuge at 800 g for 20 min. Following centrifugation, two ring-shaped, milky white cell layers will appear below the plasma layer: The upper layer contains mononuclear cells, and the lower layer contains neutrophils. Carefully transfer the neutrophil layer to a new centrifuge tube, add three to five times the volume of washing liquid, gently mix the cells, and centrifuge at 250 g for 10 min, discarding the supernatant. Repeat this step twice to ensure thorough washing of the cells. Finally, more than 80% of neutrophils were extracted, resuspended in 0.5 mL of washing solution, and adjusted to a concentration of 1 × 10^7^ cells using a cell counter. The isolated neutrophils were washed with phosphate-buffered saline (PBS), resuspended in RPMI 1640 medium containing 10% fetal bovine serum, and cultured in a humidified incubator at 37°C with 5% CO_2_.

### 2.10. MTT

Freshly isolated neutrophils were seeded at a density of 5 × 10^4^ cells per well in 96-well plates and allowed to adhere for 24 h. After 24 h, the cells were treated with varying concentrations of COS (0.5, 1, 2.5, 5, and 10 *μ*M) for 24 h [14]. After incubation, 10 *μ*L of MTT solution (5 mg/mL) was added to each well. After an additional 4 h of incubation, the culture medium was gently removed, and 100 *μ*L of dimethyl sulfoxide was added. The absorbance of the samples was measured at 570 nm. The concentrations of COS that exhibited low, medium, and high efficacy (1, 2.5, and 5 *μ*M) were selected for further experiments.

### 2.11. Induction and Intervention of NETs

Freshly isolated neutrophils were resuspended in RPMI 1640 complete medium, seeded in cell culture plates, and incubated for 1 h. Following incubation, the cells were divided into the following treatment groups:

The PMA group was treated with 100 nM PMA for 3 h to induce the formation of NETs [20].

The PMA + COSL group received both 100 nM PMA and 1 *μ*M COS for 3 h.

The PMA + COSM group was treated with 100 nM PMA and 2.5 *μ*M COS for 3 h.

The PMA + COSH group was treated with 100 nM PMA and 5 *μ*M COS for 3 h.

After the treatment period, cells from each group were collected for subsequent analysis.

### 2.12. Immunofluorescence

Paraffin-embedded renal tissue sections were subjected to immunofluorescence staining. CD41 staining was performed to assess platelet activation within the renal tissue. Double immunofluorescence staining for MPO and CitH3 was conducted to visualize NET formation. For double immunofluorescence staining to visualize NET formation, primary antibodies against MPO and CitH3 were applied simultaneously and incubated overnight at 4°C. After washing, secondary antibodies were applied and incubated for 1 h at room temperature. Nuclei were counterstained with DAPI. ImageJ software was used to quantify the fluorescence intensity and colocalization of MPO and CitH3.

For neutrophils extracted and treated in vitro, double immunofluorescence staining using SYTOX Green and CitH3 was performed to evaluate NET formation. Specifically, neutrophils were fixed with 4% paraformaldehyde for 15 min at room temperature and permeabilized with 0.1% Triton X-100 for 5 min. Cells were then blocked with 5% BSA in PBS for 1 h. Primary antibodies against CitH3 were applied and incubated overnight at 4°C. The revised statement is “After washing with PBS, secondary antibodies were applied and incubated for 1 h at room temperature. SYTOX Green was used to stain extracellular DNA and was applied for 10 min at room temperature. Nuclei were counterstained with DAPI. The fluorescence intensity of SYTOX Green was quantified using ImageJ software. NET formation was normalized to the PMA group (permeabilized cells treated with 100 nM PMA for 3 h) to account for variations in cell density and staining intensity.

### 2.13. Western Blot

Total protein and nuclear protein were extracted from renal tissues and neutrophils of each group. The extraction procedures followed the instructions provided by the respective kits. Protein concentrations were determined using the BCA assay. The extracted proteins were separated by SDS-PAGE and transferred onto PVDF membranes. The membranes were blocked with a 5% skimmed milk solution for 2 h, then incubated overnight at 4°C with the appropriate primary antibody. After three washes with TBST, the membranes were incubated with HRP-conjugated secondary antibodies for 2 h at room temperature. The membranes were then washed again and developed using ECL chemiluminescence. Protein bands were quantified using ImageJ software.

### 2.14. Statistics

Data were analyzed using SPSS 23.0 software and are presented as mean ± standard deviation (⁣^−^*x* ± *s*). Statistical graphs were generated with GraphPad Prism 8.30. One-way ANOVA was used to compare data across multiple groups, and LSD's post hoc test was applied for pairwise comparisons. A *p* value of < 0.05 was considered statistically significant.

## 3. Results

### 3.1. COS Demonstrates Therapeutic Effect on DN Mice

**Figure 1:**
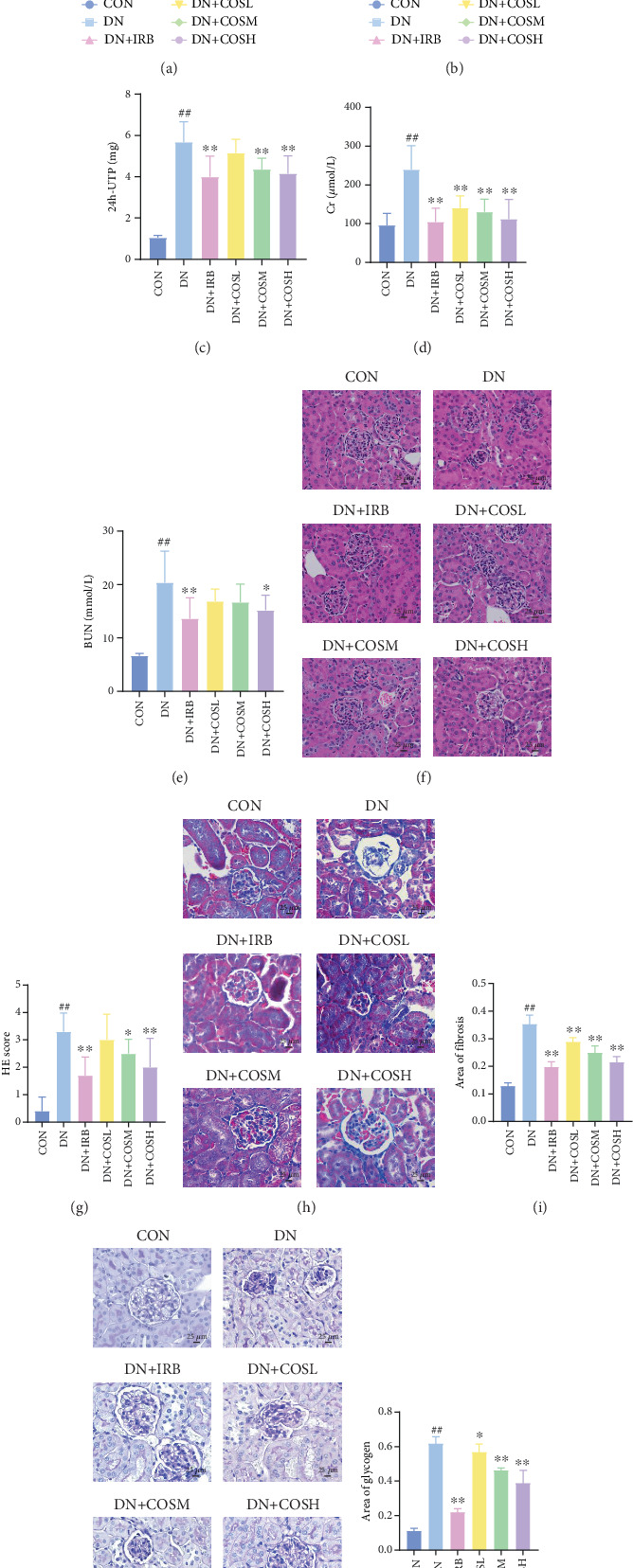
COS intervention improves renal injury in DN mice. DN mice were given COS intervention at various concentrations (10, 20, and 40 mg/kg). The degree of renal injury in each group was assessed via measurement of blood glucose, body weight, renal function, and pathological staining. (a, b) COS intervention significantly reduces (a) blood glucose and increases (b) body weight in DN mice. (c–e) COS intervention improves renal function and reduces levels of (c) 24-UTP, (d) Cr, and (e) BUN in DN mice. (f–k) COS intervention improves renal histopathological injury in DN mice. (f, g) Results of H&E staining show that COS improves renal histopathological morphology. (h, i) Masson staining shows that COS reduces renal tissue fibrosis. (j, k) PAS staining shows that COS reduces renal tissue glycogen deposition. Data are presented as means ± SD. *n* = 10 per group. ⁣^#^*p* < 0.05, ⁣^##^*p* < 0.01 versus control group; ⁣^∗^*p* < 0.05, ⁣^∗∗^*p* < 0.01 versus DN group.

Blood glucose levels showed that mice in the DN group had significantly higher blood glucose compared to the CON group. However, after COS intervention, the DN + COSH group exhibited significantly lower blood glucose (Figure 1a). Regarding body weight, mice in the DN group had significantly reduced body weight compared to the CON group, while COS treatment significantly alleviated the weight loss. The DN + COSH group displayed the most pronounced improvement in body weight (Figure 1b).

Renal function tests revealed that the 24 h-UTP content and serum levels of Cr and BUN were significantly elevated in the DN group compared to the CON group. These markers were notably reduced after COS intervention, particularly in the DN + COSH group, which showed the greatest improvement, approaching the levels seen in the positive CON group (DN + IRB) (Figures 1c, 1d, and 1e). Histological analysis by H&E staining indicated severe renal damage in the DN group compared to the CON group. This damage included glomerular hypertrophy and increased stromal hyperplasia. In contrast, renal tissue from the DN + IRB group and the DN + COS dose groups displayed varying degrees of improvement, with a reduction in glomerular hypertrophy and stromal changes (Figure 1f,g). Collagen fibers, which were more dispersed and intensely stained in the DN group, showed improved distribution and less intense staining in the DN + IRB and all COS treatment groups (Figure 1h,i). PAS staining results demonstrated that, compared to the CON group, the DN group exhibited thickened glomerular basement membranes, pronounced matrix hyperplasia in the mesangial region, darker purplish-red coloration, and increased glycogen deposition in renal tissue. In contrast, the DN + IRB group and the COS treatment groups showed reduced glomerular basement membrane thickening, less matrix hyperplasia, lighter purplish-red coloration, and lower glycogen deposition (Figure 1j,k). These findings suggest that the effect of COS in ameliorating DN is dose-dependent, with the DN + COSH group showing the most significant therapeutic effect, similar to that of the positive CON (DN + IRB). Based on these results, we selected the high-dose COS for further investigation of the underlying mechanisms in subsequent experiments.

### 3.2. Transcriptomic Analysis of the Effect of COS Intervention on Differential Genes in Renal Tissues of DN Mice

**Figure 2:**
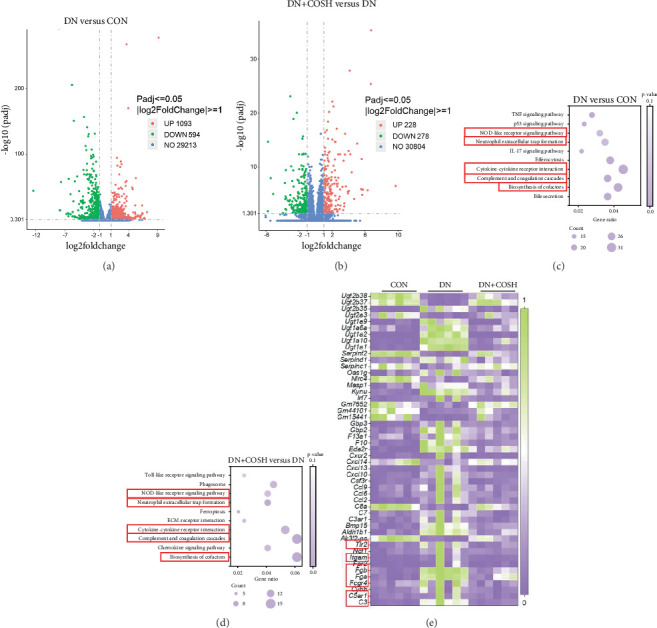
Transcriptomic analysis results from screening of differential genes after COS intervention in DN mice. Transcriptomic analysis was performed on CON, DN, and DN + COSH groups. Differential genes (DEGs) were screened for DN versus CON and DN + COSH versus DN using |log_2_(foldchange)| ≥ 1 and *p*_adj_ ≤ 0.05 as criteria, respectively. (a, b) Volcano plots visualizing DEGs. (c–e) KEGG pathway enrichment analysis revealing intersecting pathways of (c) DN versus CON and (d) DN + COSH versus DN, (e) which were enriched in Fgb, Fga, C3, Itgam, C5ar1, Tlr2, and Fcgr4. *n* = 3 per group.

We next conducted transcriptomic analysis of renal tissues from the CON, DN, and DN + COSH groups to identify DEGs between DN and CON and between DN + COSH and DN, using the criteria |log_2_(foldchange)| ≥ 1 and *p*_adj_ ≤ 0.05. The volcano plot revealed that, compared to the CON group, the DN group had 1093 upregulated genes and 594 downregulated genes. In contrast, the DN + COSH group exhibited 228 upregulated genes and 278 downregulated genes compared to the DN group (Figure 2a,b). We then performed KEGG pathway enrichment analysis on these DEGs. From the enriched pathways, we focused on those that intersected between the DN versus CON and DN + COSH versus DN comparisons. These pathways were primarily associated with inflammation and platelet activation (Figure 2c,d). Heatmap analysis of the intersecting genes within these pathways revealed that COS intervention downregulated the expression of genes related to NET formation (Figure 2e). Studies have demonstrated a significant correlation between NET formation and both inflammatory responses and platelet activation [21]. We postulate that COS may attenuate inflammatory processes and suppress platelet activation via the inhibition of NET biogenesis. Consequently, we investigated the modulatory effects of COS on both inflammatory cascades and platelet activation dynamics.

### 3.3. COS Inhibits Renal Tissue Inflammation and Platelet Activation in DN Mice

**Figure 3:**
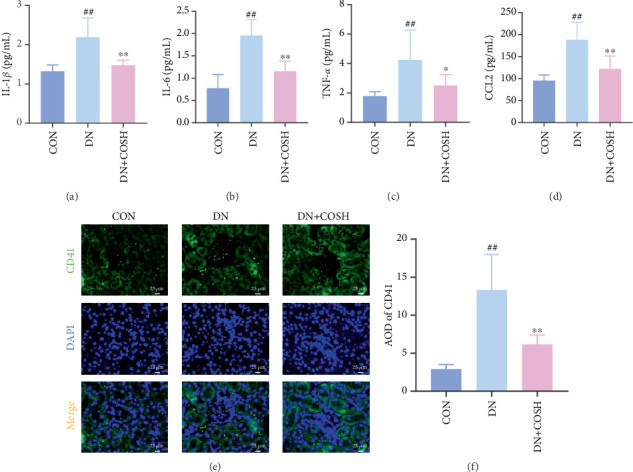
Reduction of expression of inflammation- and thrombosis-related factors via COS intervention in renal tissues of DN mice. Renal tissues of mice in CON, DN, and DN + COSH groups were collected. Levels of inflammation-related factors in renal tissues were detected by ELISA. Platelet markers in renal tissues were detected by immunofluorescence. (a–d) COS intervention significantly downregulates levels of (a) IL-1*β*, (b) IL-6, (c) TNF-*α*, and (d) CCL2 in renal tissues of DN mice and attenuates inflammatory response in DN mice. In addition, (e, f) COS intervention significantly reduces positive expression of CD41 in glomeruli of DN mice and attenuates coagulation dysfunction phenomenon in DN mice. *n* = 10 per group.

ELISA results showed that the expression of IL-1*β*, IL-6, TNF-*α*, and CCL2 was significantly elevated in the DN group compared to the CON group. In contrast, the expression of these markers decreased significantly in the DN + COSH group compared to the DN group, indicating that COS exerted a strong anti-inflammatory effect (Figures 3a, 3b, 3c, and 3d). CD41 is a well-characterized surface glycoprotein that serves as a canonical activation marker of platelets, with its expression level being closely associated with platelet aggregation dynamics [22]. Immunofluorescence staining revealed a marked increase in CD41 expression in the glomeruli of mice in the DN group compared to the CON group. Conversely, CD41 expression was significantly reduced in the glomeruli of mice in the DN + COSH group compared to the DN group (Figure 3e,f).

### 3.4. COS Intervention Reduces Expression of NET-Related Factors in Renal Tissues of DN Mice

**Figure 4:**
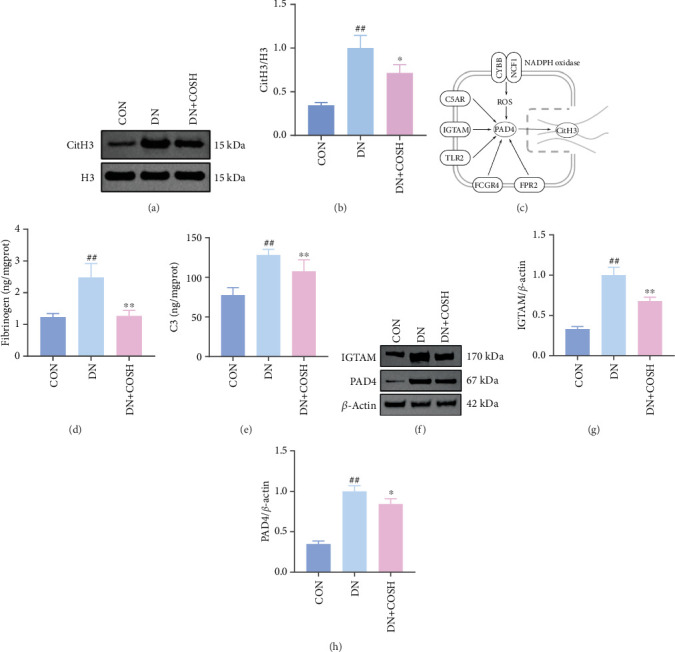
COS intervention reduces expression of DN NET-related factors in renal tissues of DN mice. Renal tissues from CON, DN, and DN + COSH mice were collected. Western blot was used to detect levels of histone guanosine. ELISA and a biochemical kit were used to detect expression of fibrinogen and C3, genes related to NET formation. Western blot was used to detect expression of IGTAM and PAD4 proteins. (a, b) COS intervention inhibits NET formation in renal tissues of DN mice given a reduction in the expression of CitH3/H3 in nuclei of renal tissue cells. (c) Expression of genes associated with NET formation was also reduced, including (d) fibrinogen and (e) C3, as well as reduced expression of proteins including (f, g) IGTAM and (f, h) PAD4. (a, b, f, g, h) *n* = 3; (d, e) *n* = 10.

Transcriptomic analysis revealed that the DEGs were closely associated with NET formation following COS intervention. Additionally, the literature reports that NET formation is a key contributor to both the inflammatory response and platelet activation–mediated thrombosis in DN [21]. Based on these findings, we next examined the effect of COS on NET formation in DN mice. We then extracted cytosolic proteins from renal tissues, and western blot analysis revealed that CitH3 expression was significantly elevated in the DN model. However, COS treatment notably reduced CitH3 protein levels (Figure 4a,b). Among the NET-related genes identified in the transcriptomic analysis, *Fgb*, *Fga*, and *C3* are ligands for *Itgam*, which can activate PAD4 through NADPH-generated ROS. Moreover, the activation of *C5ar1*, *Tlr2*, and *Fcgr4* also promotes PAD4 activation, ultimately leading to increased *CitH3* expression (Figure 4c). To further investigate this pathway, we measured fibrinogen content in mouse renal tissues using an ELISA kit. The results showed that COS significantly reduced elevated fibrinogen levels in the DN model. We also measured C3 levels in renal tissues using a biochemical kit, and the results demonstrated that COS significantly lowered the elevated C3 levels in DN mice (Figure 4d,e). Additionally, western blot analysis revealed that ITGAM and PAD4 expression were significantly increased in the DN model, but COS intervention effectively reversed this increase (Figures 4f, 4g, and 4h).

### 3.5. COS Inhibits NET Formation In Vitro

**Figure 5:**
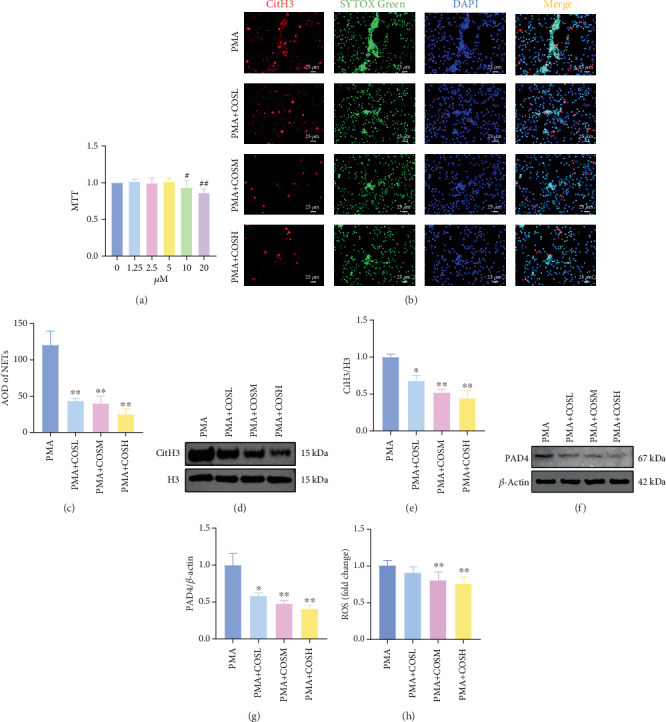
In vitro experiments showing the inhibition of NET formation by COS intervention. Mice peripheral blood neutrophils were extracted. MTT was then used to screen COS intervention dosages on (a) neutrophils. Subsequently, 100 nM PMA was used for 3 h. For in vitro validation, SYTOX Green + CitH3^+^ expression was detected by immunofluorescence, ROS level was detected by a biochemical kit, and CitH3/H3 in the nucleus and PAD4 protein expression in total protein were detected by western blot. (b–e) COS intervention reduces NET formation after PMA induction, (b, c) decreases SYTOX Green and CitH3 expression, and (d, e) decreases CitH3/H3 protein expression in nucleus proteins. COS intervention also reduces (h) ROS levels and (f, g) PAD4 protein expression in cells. (a) *n* = 6; (b–g) *n* = 3; (h) *n* = 6.

The inhibitory effect of COS on NET formation was further validated in vitro. We extracted peripheral blood neutrophils from mice and screened COS doses (1, 2.5, and 5 *μ*M) as low, medium, and high doses, respectively, using MTT for dose selection (Figure 5a). To induce NET formation, we treated the neutrophils with PMA and administered COS at the selected doses. To confirm PMA's effectiveness in inducing NETs, SYTOX Green and CitH3 expression levels and ROS levels were compared between normal and PMA-treated groups. The results showed that PMA induction increased SYTOX Green and CitH3 expression, along with ROS levels (Figure S1). Immunofluorescence analysis showed that COS treatment, at all doses, reduced the expression of SYTOX Green and CitH3 after PMA induction, with varying degrees of inhibition across the dose groups (Figure 5b,c). Additionally, COS treatment decreased CitH3 protein levels in the nuclear extracts from all groups, indicating that COS effectively inhibited NET activation following PMA induction (Figure 5d,e). ROS assays demonstrated that COS also reduced ROS levels after PMA induction (Figure 5h). Western blot analysis further revealed that COS treatment significantly reduced PAD4 protein expression following PMA stimulation (Figure 5f,g). These in vitro results collectively confirm that COS can inhibit the formation of NETs in neutrophils.

## 4. Discussion

DN is the most prevalent microvascular complication in patients with diabetes mellitus. Its pathogenesis involves chronic inflammation, oxidative stress, and coagulation disorders, which interact to aggravate renal tissue injury and fibrosis [23]. Despite the availability of major treatments for DN, such as renin–angiotensin system inhibitors and glucose-control medications, these therapies do not fully prevent disease progression [24, 25]. This highlights the need for a deeper understanding of the underlying mechanisms of DN and the identification of potential therapeutic targets.

In this study, we developed a DN mouse model using a combination of a high-sugar, high-fat diet chow feeding and STZ intraperitoneal injection, a widely established method in DN research [17]. As expected, the mice in the DN group exhibited hyperglycemia and abnormal renal function, accompanied by significant histological damage, including collagen and glycogen deposition, as observed in pathological staining. These findings were consistent with the typical pathological changes associated with DN, confirming the successful induction of DN in the mice [26]. Following COS intervention, we observed a reduction in blood glucose levels in the mice, along with improvements in various biochemical indices. Pathological examination further revealed that COS had a protective effect on renal tissue, ameliorating the histopathological alterations seen in the DN mice. In this study, IRB was selected as the positive control drug, given its established clinical efficacy [27]. The results indicated no significant difference between COSH and IRB in terms of improving renal function. This finding suggests that COS is effective in treating DN and could serve as a potential alternative to IRB for DN management.

Transcriptomic analysis revealed that COS intervention significantly regulated several inflammation- and coagulation-related pathways in the DN model, including the complement and coagulation cascades, biosynthesis of cofactors, cytokine–cytokine receptor interaction, NET formation, and NOD-like receptor signaling pathway. These pathways are involved in inflammation and platelet activation–mediated thrombosis in DN. To further validate the effects of COS on inflammation and platelet activation–mediated thrombosis, we examined key indicators of these processes, such as IL-1*β*, IL-6, TNF-*α*, CCL2, and CD41. The results demonstrated that COS significantly reduced the expression of these inflammatory markers in renal tissues and also decreased the positive expression of CD41 in the glomeruli. CD41, a marker of platelet activation, plays a crucial role in thrombosis. When vascular endothelium is damaged, platelets adhere to subendothelial matrix components via CD41 [28]. This interaction triggers platelet activation, causing the release of ADP and thromboxane A2, which further amplify platelet recruitment and aggregation. Activated CD41 binds fibrinogen, promoting platelet cross-linking and clot formation [29]. These findings suggest that COS effectively alleviates both inflammation and platelet activation–mediated thrombosis in DN.

The formation of NETs is closely associated with inflammation and platelet activation–mediated thrombosis. We hypothesized that COS inhibits both inflammation and thrombosis by suppressing NET formation. The process of NET formation is strongly linked to the citrullination of histone H3, with CitH3 serving as a key biomarker for NET formation. Additionally, MPO, a major component of NET degradation products, is commonly used to assess NET formation and activity [30, 31]. To investigate the effect of COS on NET formation, we isolated renal tissue cell nuclei and applied western blot analysis to quantify CitH3 levels. The results revealed that COS significantly inhibited NET formation. Furthermore, western blot analysis of renal tissue nuclear proteins demonstrated a significant decrease in the CitH3/H3 ratio. Collectively, these findings suggest that COS effectively inhibits NET formation in vivo.

Transcriptomic analysis further indicated that COS regulates several key genes involved in NET formation, including *Fgb*, *Fga*, and *C3*. *Fgb* and *Fga* encode fibrinogen, a central component of the coagulation cascade. Fibrinogen not only plays a critical role in thrombosis [32] but also mediates neutrophil activation through its binding to Itgam/CD11b [33]. The activation of Itgam recruits the NADPH oxidase complex, promoting the production of ROS, which in turn activates PAD4. This cascade triggers histone citrullination, ultimately leading to NET formation [34]. The significant upregulation of *C3* gene expression is a key marker of aberrant complement system activation in the DN model. Cleavage products of *C3*, such as *C3a* and *C3b*, amplify inflammatory responses and accelerate granulocyte activation and degranulation by binding to Itgam/CD11b on neutrophil surfaces [35]. In our study, we observed that COS significantly suppressed the expression of *Fgb*, *Fga*, and *C3*, suggesting that this regulation of the complement system may play a critical role in inhibiting NET formation.

Beyond these genes, COS intervention also significantly reduced the expression of *C5ar1*, *Tlr2*, and *Fcgr4*. The activation of *C5ar1* is mediated by complement *C5a*, a potent chemotactic molecule generated during complement activation. *C5a* recruits neutrophils and induces their release of NETs [36]. Similarly, *Tlr2*, a critical pattern recognition receptor on neutrophil surfaces, recognizes pathogen-associated or injury-associated molecular patterns. This recognition activates downstream signaling pathways, such as MEK/ERK, promoting inflammation and PAD4 activation [37, 38]. Lastly, *Fcgr4*, a member of the Fc*γ* receptor family, binds to immune complexes, enhancing neutrophil degranulation and activation. This process further drives NET release [39]. Together, these findings suggest that COS effectively regulates the expression of key genes associated with complement activation and neutrophil function, thereby mitigating NET formation and inflammation in DN.

*Fcgr4*, a member of the Fc*γ* receptor family, enhances neutrophil degranulation and activation by binding to immune complexes, which in turn promotes NET release. Notably, these genes and signaling pathways may interact synergistically. For instance, complement system activation not only drives NET formation through the downstream effects of C3 and C5a but also amplifies inflammation by enhancing Tlr2 and Fcgr4 signaling [40]. C3 and C5a facilitate granulocyte adhesion and chemotaxis by binding to Itgam, which accelerates the recruitment of NADPH oxidase complexes. This recruitment increases ROS production, ultimately activating PAD4 and promoting NET formation [41, 42]. Our experiments demonstrated that COS intervention significantly downregulated the expression of these genes and reduced PAD4 protein levels in renal tissues. These findings suggest that COS inhibits NET formation through multiple mechanisms, including the suppression of complement activation and the inhibition of Tlr2- and Fcgr4-mediated inflammatory signaling.

**Figure 6:**
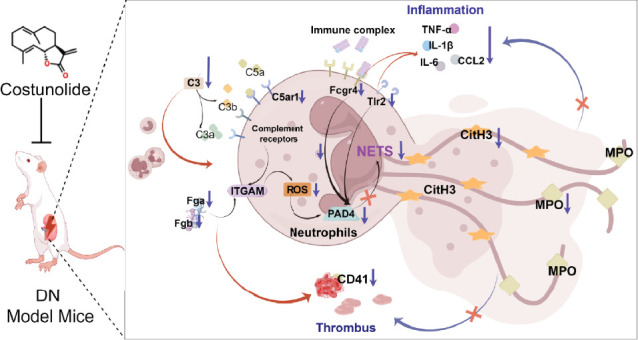
COS alleviates inflammation and renal thrombosis in DN mice, potentially by inhibiting excessive NET formation.

To further validate the mechanism of action of COS, we conducted in vitro experiments using PMA, a commonly used inducer of NET formation. Previous studies have demonstrated that 100 nM PMA induces significant NET formation after 3 h of in vitro treatment [20]. Consistent with these reports, our results showed an elevated expression of NET markers in neutrophils following 3 h of PMA treatment, confirming successful NET induction. Furthermore, COS intervention significantly reduced the expression of SYTOX Green and CitH3, as well as the PAD4 protein levels. COS also markedly inhibited the PMA-induced increase in ROS production. These findings were in agreement with the results of our in vivo experiments and further support the mechanism by which COS inhibits NET formation through multiple pathways (Figure 6).

Recent evidence has implicated an intricate, bidirectional communication between platelets and NETs in various inflammatory conditions. NETs promote thrombin generation [43], and, in turn, activated platelets promote NET formation [44] which are proposed to occur most likely through neutrophil–platelet interaction mediated by P-selectin [45]. Platelets activate neutrophil integrins and drive NETs via P-selectin [46, 47], while COS reduces P-selectin expression to ease inflammation [48]. In addition, TLR4 activation prompts ROS generation, which in turn activates the NLRP3 inflammasome and Caspase 1. Once activated, Caspase 1 triggers the cleavage of GSDMD, inducing pyroptosis in platelets. During pyroptosis, oxidized mitochondrial DNA is released and serves as a DAMP to further promote NET formation. Moreover, the upstream signal activates Akt, which in turn activates mTOR. mTOR promotes the expression of PADI4 by regulating the downstream signal pathway. PADI4 induces chromatin decondensation and release by catalytic histone modification, which ultimately leads to the formation of NET. However, COS can alleviate inflammatory responses by downregulating TLR4 expression [49] and inhibiting the Akt/mTOR pathway [50]. This highlights the potential mechanisms of COS alone in inhibiting NETs. Future research should verify this through additional experiments to confirm the effect of COS treatment on NET inhibition without glucose modulation.

In conclusion, this study is the first to systematically demonstrate the role of COS in alleviating DN by inhibiting NET formation and to identify potential molecular mechanisms. These findings not only confirm the therapeutic potential of NET pathway modulation in DN but also identify COS as a promising agent for DN management. Future research could further explore the molecular targets of COS and its potential clinical applications.

## Data Availability

The data that support the findings of this study are available from the corresponding author upon reasonable request.
