# Supplementary material for: Costunolide Reduces DN Inflammatory Response and Renal Thrombosis by Inhibiting NET Formation
Source: J Diabetes Res. 2025 Jul 17;2025:1159325. doi: 10.1155/jdr/1159325 (PMC12289368; doi:10.1155/jdr/1159325)
Supplement: Supporting Information — Additional supporting information can be found online in the Supporting Information section. Data S1: The details of materials and reagents. [file 1159325.f1.docx]

***Supplementary Material***

**Reagents**

Streptozotocin (S17049), irbesartan (S42406) were purchased from Shanghai yuanye Bio-Technology Co., Ltd. (Shanghai, China). 24h-UTP(C035-2-1), creatinine (Cr, C011-2-1) and blood urea nitrogen (BUN, C013-2-1), ROS ([E004-1-1](http://www.njjcbio.com/products.asp?id=819)), BCA(A045-4-2), HE staining assay kit ([D006-1-1](http://www.njjcbio.com/products.asp?id=476)), Masson ([D026-1-3](http://www.njjcbio.com/products.asp?id=529)), PAS (D004-1-1) were purchased from Nanjing Jiancheng Biological Engineering Institute (Nanjing, China). The kit used for neutrophil isolation (LZS1091) was purchased from Tianjin Haoyang Biotechnology Co., Ltd. (Tianjin , China). IL-1β(ml106733), IL-6(ml098430), TNF-α(ml002095), Ccl2(ml063804)、C3(ml106691), Fibrinogen(ml098415) were purchased from Shanghai meilian Bio-Technology Co., Ltd. (Shanghai, China).CD41(ab134131, 1/100), MPO (ab208670, 1/100), CitH3(ab219407, 1/100) were purchased from Abcam (Shanghai, China). SYTOX Green(C1181S) were purchased from Beyotime Biotechnology (Shanghai, China). IGTAM (93169S, 1/1000) were purchased from cell signaling technology (Shanghai, China). PAD4(17373-1-AP, 1/6000) were purchased from proteintech (Wuhan, China). H3(ab1791, 1/5000), CitH3(ab219407, 1/1000) were purchased from Abcam (Shanghai, China).

**
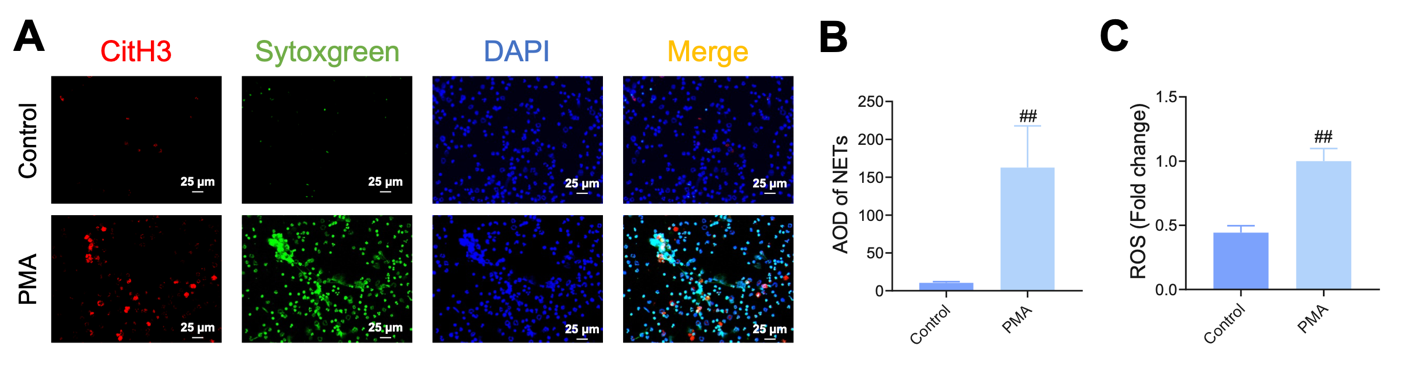
**

**FigureS1 PMA induction increased the expression of Sytox Green and CitH, along with ROS levels.**
